# The effect of a very brief smoking-reduction intervention in smokers who have no intention to quit: Study protocol for a randomized controlled trial

**DOI:** 10.1186/s12889-015-1749-7

**Published:** 2015-04-25

**Authors:** Lei Wu, Yao He, Bin Jiang, Di Zhang, Hui Tian, Fang Zuo, Tai Hing Lam, Yee Tak Derek Cheung

**Affiliations:** Institute of Geriatrics, Chinese PLA General Hospital, 28 Fuxing Road, Beijing, 100853 China; Beijing Key Laboratory of Aging and Geriatrics, Chinese PLA General Hospital, 28 Fuxing Road, Beijing, 100853 China; State Key Laboratory of Kidney Disease, Chinese PLA General Hospital, 28 Fuxing Road, Beijing, 100853 China; Department of Acupuncture, Chinese PLA General Hospital, 28 Fuxing Road, Beijing, 100853 China; Department of Endocrinology, Chinese PLA General Hospital, 28 Fuxing Road, Beijing, 100853 China; School of Public Health, The University of Hong Kong, Pok Fu Lam, Hong Kong; School of Public Health, Li Ka Shang Faculty of Medicine, The University of Hong Kong, 5/F William MW Mong Block, Faculty of Medicine Building, 21 Sassoon Road, Pok Fu Lam, Hong Kong

**Keywords:** Very brief counseling, Smoking-reduction intervention, No intention to quit, Randomized controlled trial, Telephone follow-up

## Abstract

**Background:**

Tobacco use is one of the most common preventable causes of death, but more than half of the Chinese men still use tobacco products. Moreover, 63.6% of Chinese smokers have stated that they would not consider quitting. Specialized and intensive smoking-cessation services are too expensive and passive to have major clinical and public health impacts in developing countries like China. Smoking cessation medications are not covered by medical insurance, and their high price prevents Chinese smokers from using them. Brief interventions are needed to provide cost-effective and timesaving tobacco dependence treatments in China mainland.

**Methods/design:**

We describe a two-arm randomized controlled trial for smokers who have no intention to quit. The project will be conducted in outpatient clinics at a large hospital in Beijing, China. Both arms include one face-to-face interview plus five follow-up interventions. Each intervention will last approximately one minute. Subjects allocated to the smoking-reduction intervention arm (SRI) will be advised to reduce smoking consumption to at least half of their current consumption level within the next month. All subjects in the SRI will be warned to bear in mind that an attempt to reduce smoking is an intermediate step before complete cessation. Smokers who have successfully reduced their smoking consumption will be encouraged to completely cease smoking. Controls are subjects allocated to the exercise- and diet-advice arm (EDA) and will be given advice about healthy diet and physical activity, but the advice will not include smoking cessation or reduction. Data collection will be done at baseline and at each follow-up interview using standardized questionnaires. The primary outcomes include self-reported and biochemically verified 7-day point prevalence and prolonged abstinence rates at 12-month follow-up.

**Discussion:**

We expect that an intention to quit in smoking outpatients can be motivated by physicians in the clinic setting. If this very brief smoking-reduction intervention can be demonstrated to have a positive impact on long-term smoking cessation, this strategy has the potential to be a viable and acceptable approach and may be used widely in China and elsewhere.

**Clinical trial registration:**

ClinicalTrials.gov: NCT02370147 (date of registration: 23th February, 2015).

## Background

Tobacco use is one of the most common preventable causes of death, and many countries are expending effort to reduce the smoking prevalence. However, the prevalence of smoking in China has declined little in recent years. The Global Adult Survey reported that the prevalence of smoking among Chinese men decreased from 57.4% in 2002 to 52.9% in 2010 [1,2]; thus, more than half of Chinese men are still current smokers. Although quitting can reduce the mortality due to smoking-induced diseases, 63.6% of Chinese smokers would not consider quitting [2]. Therefore, it is critical to expand smoking prevention and cessation efforts in China.

In developed countries and regions, including Hong Kong and Taiwan with mostly Chinese [3-6], smoking cessation services, such as smoking cessation clinics and telephone quitlines, are well established. These services play an important role in public health. However, these specialized and intensive smoking-cessation services are too expensive and passive to have major clinical and public health impacts in developing countries like China, as they require continuous and enormous funding and intensive training for counselors, and involve other costs, such as pharmacotherapy. Pharmacotherapy is an effective treatment modality for tobacco dependence, but it is not widely used in China mainland [7,8]. One of the most important reasons why pharmacotherapy is not widely used for smoking cessation is that smoking-cessation medications are not covered by medical insurance, and they are too expensive. Hence, there is an urgent need for a much cheaper yet effective strategy for helping smokers to quit.

In general, commonly used behavioral intervention categories fall into two categories: brief or minimal interventions and intensive interventions [9]. A brief or minimal intervention was defined in a Cochrane review as less than 20 minutes of advice plus up to one follow-up session [10]; other interventions were defined as intensive. Stead et al. reported that the quit rate following brief advice from physicians was significantly higher than that following no advice (or usual care); the relative risk (RR) was 1.66, and the 95% confidence interval (CI) was 1.42 to 1.94 [10]. However, the quit rates following intensive counseling were not found to be significantly different from those following brief counseling in two Cochrane reviews [10,11]. As a result, brief behavioral interventions are recommended to address the need for cost-effective and timesaving interventions for smokers [12]. In 2013, Lin et al. reported that their pilot randomized controlled trial in Guangzhou, China showed that a 30-second smoking cessation intervention by a physician was an effective way to encourage smokers to quit; the 12-month continuous abstinence rate was 14.9%, compared with 3.8% in the control group. These results suggest that a very brief intervention can achieve a measurable effect in China mainland [13,14].

For smokers who have no intention to quit, smoking-reduction interventions may be a therapeutic choice [15,16]. The common definition of successful smoking reduction is a self-reported reduction of daily cigarette consumption by 50% or more compared with baseline [17]. However, smoking-reduction treatment has been highly controversial in the past [18,19]. The main concerns and uncertainties regarding this approach were that (a) a short-term reduction may not be maintained for long periods [20]; (b) smoking reduction does not necessarily imply harm reduction [21-23]; and (c) future attempts to quit smoking may be undermined by short-term reductions in daily cigarette consumption [20]. However, according to a meta-analysis by Asfar et al., pharmacological combined with smoking-reduction interventions significantly increased long-term smoking cessation rates by factors of 2.14 [19]. Recently, a randomized controlled trial by Chan et al. also supported this conclusion [24]. It has been reported that a smoking-reduction approach may be more likely to be adopted by a broader range of smokers than traditional smoking-cessation interventions [25]. However, limited evidence exists about the effectiveness of behavioral smoking-reduction randomized controlled trials for smokers who have no intention to quit. Glasgow et al. implemented an individual behavioral smoking-reduction intervention for smokers who were not interested in quitting smoking, and the results favored the intervention but did not reach statistical significance [26]. To our knowledge, no previous study has investigated whether a very brief smoking-reduction intervention can increase quitting rates in China; whether this approach is feasible and effective for increasing long-term quit rates among smokers who have no intention to quit remains unclear.

Brief interventions are needed to provide cost-effective and timesaving treatments for tobacco dependence, particularly in developing countries like China. The protocol described here is designed to fit the real life conditions of China mainland. Smoking information should be routinely collected from outpatients in clinics; this interaction provides a good opportunity for doctors to persuade outpatients and their companions to quit smoking. A very brief (i.e., approximately one-minute) smoking-reduction intervention will be proposed to smokers who have no intention to quit. All smokers receiving such intervention will be asked to bear in mind that an attempt to reduce smoking is an intermediate step before complete cessation. During follow-up interviews, if smokers have already reduced their tobacco consumption, they will be encouraged to quit smoking during follow-up interviews. This very brief, active and minimal cost individual smoking reduction intervention will provide strong evidence for a new approach for treating tobacco dependence in China mainland and elsewhere.

## Methods/Design

### Study design

In the present protocol, we describe a two-arm randomized controlled trial for smokers who have no intention to quit.

### Participants, setting and procedure

The project will be conducted in the outpatient clinics of the People's Liberation Army General Hospital in Beijing, China. Physicians in outpatient clinics in the endocrinology and acupuncture departments will be asked if they want to participate in the trial. Those who agree to participate will be recruited into the current project. Before the trial starts, the participating physicians will be trained. Most of the physicians are in busy clinical settings, and they may not be able to afford the extra few minutes needed to advise each smoker to quit [14]; therefore, students of the physicians will also be trained to assist with completing the interviews.

Current smokers who are outpatients attending the clinics and their companions will be included in the project. Each self-reported smoker will be asked, “Do you have an intention to quit smoking?” Smokers who answer that they are “beginning to quit within the next 7 days” or “within 30 days” will be excluded from the trial. Smokers who answer that they intend to quit “in the next 6 months” or “after 6 months” or that “I’m not making a decision about quitting smoking” will be included in the trial [27,28].

Participants will be eligible for the study if they meet the following criteria: (1) aged ≥18 years; (2) have smoked ≥10 cigarettes per day in the past month; (3) have no intention to quit smoking; (4) agree to offer their telephone numbers and sign informed consent forms for participation.

Participants will be excluded from the study if they meet the following criteria: (1) have smoked fewer than 10 cigarettes per day in the past month; (2) have a disease that makes a physician think it would be unethical not to advise a patient to quit smoking as soon as possible; (3) are cognitively or otherwise impaired (i.e., deaf or unable to understand and complete a questionnaire reliably); (4) are pregnant or lactating females.

Signed, informed consent will be obtained from all eligible participants. Information about the project aims, interventions, assessments and data collection will be included on a paper form. Eligible participants will also be asked for a telephone number for follow-up interviews.

The study has been approved by the Independent Ethics Committee of the Chinese People's Liberation Army General Hospital.

### Data collection

Data collection will be conducted at the first visit and at each follow-up interview using standardized, structured questionnaires. The questionnaires have been developed based on questionnaires used in Hong Kong [3,29].

Baseline data will be collected during the first visit via a face-to-face interview. The collected data will include demographic characteristics (gender, age, marital status and educational level); self-reported perceived health status over the past several days (very good, good, fair, poor, very poor); smoking history (smoking status, the age at starting smoking regularly and the average number of cigarettes smoked per day); the six questions regarding nicotine dependence levels based on the Fagerström Test for Nicotine Dependence (FTND) [30]; past quitting history (whether attempts have ever been made to quit, past use of cessation products and causes of relapse); and whether the participant has any doctor diagnosed chronic diseases. Body weight will be measured in kilograms (shoes and heavy clothing will be removed, and one kilogram will be deducted for the participant’s remaining garments), and exhaled carbon monoxide level will be measured using a “Bedfont Micro II Smokerlizer” [3].

Follow-up data will be collected via telephone interviews at 1 week and 1, 3, 6 and 12 months after the first interview; questions will address smoking or smoking-reduction status, quitting attempts and withdrawal symptoms. Participants who report having abstained from smoking for more than 7 days at the 12-month follow-up will be invited to do a face-to-face biochemical verification (i.e., exhaled carbon monoxide level will be measured).

### Randomization

Before participant recruitment, a research assistant on the project will generate random numbers for group assignments using a computer. If an individual is eligible to participate in the study, and written consent is obtained, trained counselors will randomly allocate the participant to a treatment group by opening a serially labeled, opaque and sealed envelope, and a card inside will indicate the group to which the participant should be randomly allocated.

### Blinding

Given the nature of the intervention, it will be impossible to blind the subjects to their group assignments. However, the trained counselors who will conduct the telephone follow-up interviews will be different from the ones who completed the first interview and will not know the aim of the subjects; they will therefore be able to record outcome measures with minimal subjective bias. The researchers who will conduct the data analysis will be blinded to any potential identifiers.

### Sample-size calculation

Because no similar studies have previously been conducted in China mainland, the effect-size estimation is based on results from Hong Kong [14,24]. We assume that the quit rate at the 12-month follow-up of the SRI group would be 15% versus 5% for the control group. To detect a difference of 10%, it is estimated that 138 subjects would be needed for each group given an alpha level of 5% (two-sided) and a statistical power of 80%. A total of 331 participants will be recruited based on a possible loss of 20% of the participants at the 12-month follow-up.

### Description of the intervention

**Figure 1:**
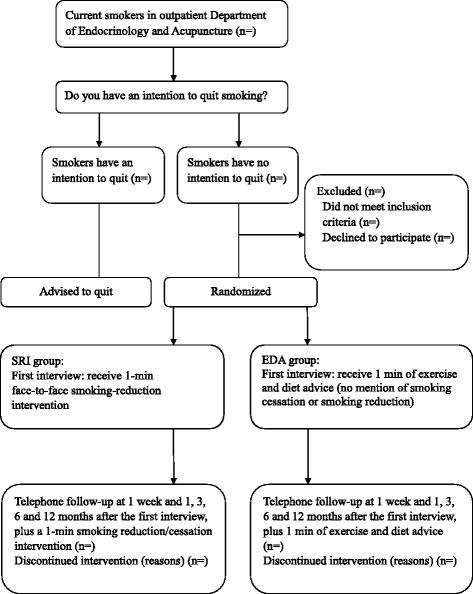
Study design. SRI: smoking-reduction intervention group; EDA: exercise- and diet-advice group.

First, smokers who have an intention to quit will be advised to quit immediately. Trained counselors will randomly allocate the smokers who have no intention to quit into two groups: the smoking-reduction intervention group (SRI) or the exercise- and diet-advice group (EDA). The study design of the current study is shown in Figure 1.

### Smoking-reduction intervention group (SRI)

In the first face-to-face interview, trained counselors will give each smoker a very brief smoking-reduction intervention lasting approximately one minute. Smokers will be warned that many non-communicable diseases are caused by smoking, such as cancer, coronary heart disease, respiratory diseases and many other health problems. Moreover, one out of two smokers will be killed by smoking [31]. Smokers will be advised to reduce smoking consumption to at least half of their total consumption within the next month. Finally, all smokers will be asked to bear in mind that the current attempt to reduce smoking would be an intermediate step before complete cessation.

The tobacco-use status of each smoker will be assessed by another trained counselor who will not know the aim of the project at 1 week and 1, 3, 6 and 12 months via telephone follow-ups. Additional smoking cessation interventions will be given at each follow-up according to their updated smoking status. Smokers will be encouraged to reduce their smoking consumption if they have not changed their smoking status, and smokers who have successfully reduced their smoking consumption will be encouraged to quit completely; smokers who have successfully quit smoking will be supported to maintain abstinence. Each follow-up will last for about one minute.

### Exercise- and diet-advice group (EDA, Controls)

In the first face-to-face interview, trained counselors will give each smoker very brief advice for about one minute. Smokers will be advised to engage in regular physical activity three or four times per week and to have a healthy and balanced diet including more fruits and vegetables, which are rich in vitamins, high in nutrition values and yet low in calories.

The tobacco-use status of each smoker will be assessed at each follow-up interview, similar to the SRI group. Smokers will be advised to engage in regular physical activity and to eat more fruits and vegetables. Each follow-up will last approximately one minute. Throughout the process, smoking cessation or smoking reduction will not be mentioned.

### Outcome assessments

The primary outcomes include self-reported and biochemically verified 7-day point prevalence and prolonged abstinence rates at 12-month follow-up interview. Participants who report being abstinent from smoking for more than seven days will be invited to complete an exhaled carbon monoxide and saliva-content (measured by NicAlert) test at the 12-month follow-up. A previous study in Hong Kong [32] suggests that few (i.e., approximately 10%) smokers will return for this test. As a result, we will use self-reported quit rates as the primary outcome measure if few smokers return.

The secondary outcome measures include smoking-reduction rates (i.e., the number of cigarettes smoked per day reduced by at least 50% compared to the baseline) and predictors of successful quitting at the 12-month follow-up, and at earlier follow-ups.

### Data analysis

Epidata (3.1) will be used to perform double entry of the collected data. We will use SPSS (IBM Corp. Armonk, NY, USA) for Windows (version 19.0) for data analysis. Baseline characteristics will be compared using chi-squared tests, Mann-Whitney tests and t tests. Regression models will be used to compare the outcomes of different interventions. We will adjust for baseline differences by including the variables in the regression models if necessary. Smokers who cannot be contacted during follow-up will be considered non-quitters or non-reducers in an intention-to-treat (ITT) analysis. For sensitivity analysis, subjects lost to follow-up will be excluded for per protocol (PP) sensitivity analyses.

## Discussion

The current protocol describes a two-arm randomized controlled trial. We aim to test the long-term (1 year) effectiveness of a very brief behavioral smoking reduction intervention on smokers who have no intention to quit.

Previous studies have used various definitions and time frames to define an intention to quit, such as ‘not willing to quit in the next four weeks’ [24], “uninterested in quitting at that time” [26], “no intention to quit within the next month” [33] or “not ready to quit” [34]. In the current trial, we use a recognized theory that incorporates a stage-of-change model (include pre-contemplation, contemplation, preparation, action and maintenance) [19,27,28]. We define pre-contemplators (will quit after 6 months or undecided about quitting) and contemplators (will quit in the next 6 months) as “having no intention to quit.”

While it has been reported that few smokers have an intention to quit in China [35], physicians should play an important role in smoking cessation [36,37]. Physicians have a good opportunity to advise outpatients when they visit their clinics. We expect that more outpatients can be motivated to quit by physicians in clinic settings. Traditional smoking-cessation services often focus on smokers who express an interest in quitting. Clearly, it will be more effective for physicians to offer assistance to all smokers, not only to those selected by motivation [38]. Different methods of intervention should be used for smokers with different smoking histories. If a very brief smoking-reduction intervention is demonstrated to have a positive impact on long-term smoking cessation, this strategy has the potential to be a viable and acceptable approach that could be used widely in China and elsewhere.
